# Epidemiological survey on canine parvovirus in the Urban Commune of Labé, Guinea

**DOI:** 10.1186/s40575-026-00154-5

**Published:** 2026-07-01

**Authors:** Noumouké Kante, Lanceï Kaba, Madou Sanou, Fatoumata Binta Barry, Nafissatou Ouedraogo, Almamy Ousmane Deen Camara, Aboubacar Demba Camara, Yacouba Konate, Foromo Félix Haba, Alpha Oumar Sily Diallo, Youssouf Sidime, Nicolas Barro, Abdoulaye Kuan Traore

**Affiliations:** 1 Department of Veterinary Medicine, Higher Institute of Veterinary Sciences and Medicine (ISSMV) of Dalaba, Dalaba, Guinea; 2 Joseph KI-Zerbo University, Ouagadougou, Burkina Faso; 3 Norbert Zongo University, Koudougou, Burkina Faso; 4 Daniel Ouezzin COULIBALY University, Ouagadougou, Burkina Faso

**Keywords:** Canine parvovirus, CPV-2, Prevalence, PCR, Risk factors, Guinea

## Abstract

Canine parvovirus (CPV-2) is one of the most important viral diseases affecting domestic dogs worldwide, particularly in unvaccinated and poorly managed canine populations. Despite its major veterinary and economic impact, epidemiological information regarding canine parvovirus remains scarce in Guinea, especially in the urban commune of Labé. This study aimed to determine the prevalence of canine parvovirus and identify factors associated with infection among dogs in the study area. A descriptive, analytical, cross-sectional study was conducted between September and November 2024 among 102 dogs selected from seven neighborhoods of the urban commune of Labé. Rectal swab samples were collected from dogs after owner consent and analyzed using real-time polymerase chain reaction (qPCR) for the detection of CPV-2 genetic material. Epidemiological and management data were collected using structured questionnaires administered to dog owners and breeders. Statistical analyses were performed using logistic regression to evaluate potential associations between infection and selected risk factors. The overall prevalence of canine parvovirus was 10.8% (11/102), confirming active viral circulation in the study area. Higher prevalence rates were observed in male dogs (11.9%) and free-roaming dogs (11.5%) compared with female dogs and caged dogs, respectively. Dogs aged 6–10 years showed the highest prevalence (20.0%). However, no statistically significant association was identified between infection and the investigated factors (*p* > 0.05). In addition, the survey revealed low owner awareness of canine parvovirus and limited vaccination practices. These findings indicate that canine parvovirus remains an important animal health concern in the urban commune of Labé. Strengthening vaccination programs, improving owner awareness, promoting responsible dog management, and reinforcing epidemiological surveillance are necessary to reduce viral transmission and associated canine health losses.

## Simple summary

Canine parvovirus (CPV-2) is a highly contagious viral disease affecting dogs worldwide, particularly unvaccinated animals. In Guinea, epidemiological information regarding this disease remains limited, especially in the urban commune of Labé. This study aimed to determine the prevalence of canine parvovirus and identify associated risk factors among dogs living in the study area. A cross-sectional survey was conducted among 102 dogs from seven neighborhoods of Labé between September and November 2024. Rectal swab samples were collected and analyzed using real-time polymerase chain reaction (qPCR) techniques for the detection of canine parvovirus DNA. Information regarding dog management practices and owner awareness of the disease was also collected through questionnaires. The overall prevalence of canine parvovirus was 10.8%, indicating active viral circulation in the area. Higher prevalence rates were observed in male dogs, adult dogs, and free-roaming animals, although no statistically significant association was identified between infection and the investigated factors. The survey also revealed limited owner awareness and insufficient vaccination practices. These findings highlight the need to strengthen vaccination programs, improve public awareness, and reinforce epidemiological surveillance to reduce the circulation of canine parvovirus in Guinea.

## Introduction

Canine parvovirus (CPV-2) is a highly contagious viral disease that constitutes a major challenge for canine health worldwide. Since its first identification in the late 1970s, the virus has rapidly spread across Europe, North America, Asia, Africa, and Australia, becoming one of the most important infectious diseases affecting domestic dogs [4]. The disease is caused by Canine Parvovirus type 2, a small non-enveloped DNA virus belonging to the family Parvoviridae. Several antigenic variants, particularly CPV-2a, CPV-2b, and CPV-2c, have subsequently emerged and continue to circulate globally [7].

Clinically, canine parvovirus mainly affects puppies and unvaccinated dogs, although adult animals may also become infected. The disease is generally characterized by hemorrhagic diarrhea, vomiting, fever, anorexia, dehydration, lethargy, and leukopenia, which may lead to severe gastroenteritis and death in the absence of rapid treatment [3]. Transmission occurs primarily through the fecal–oral route, and the virus is particularly feared because of its high resistance in the environment and its ability to remain infectious for prolonged periods [4].

Despite the availability of effective vaccines, canine parvovirus remains endemic in many developing countries where vaccination coverage is insufficient and access to veterinary services is limited. In such contexts, free-roaming dog populations, poor hygiene conditions, uncontrolled breeding practices, and low owner awareness contribute significantly to viral circulation [6]. Studies conducted in different countries have reported variable prevalence rates depending on geographical conditions, diagnostic techniques, vaccination status, and dog management practices. In southern Brazil, Oliveira et al., [8] reported significant circulation of CPV-2c associated with important clinical and epidemiological impacts. Similarly, Chabchoub et al., [2] documented active canine parvovirus infections in Tunisia, while Kadiri and Amrani [5] highlighted the persistence of the disease in Morocco due to insufficient preventive measures and limited public awareness.

In Africa, epidemiological information regarding canine parvovirus remains relatively scarce compared with data available from Europe and North America. This lack of surveillance data limits understanding of viral circulation patterns and hinders the implementation of effective prevention and control programs. In Guinea, and particularly in the urban commune of Labé, information concerning the epidemiology of canine parvovirus is extremely limited, despite frequent reports of suspected clinical cases by veterinarians and dog owners. The absence of structured epidemiological surveillance systems, combined with inadequate vaccination coverage and increasing dog mobility, may favor the persistence and spread of the virus within canine populations.

Molecular diagnostic methods such as polymerase chain reaction (PCR) are currently considered highly sensitive and specific tools for the detection of CPV-2 genetic material [4]. Compared with rapid antigen detection tests, PCR provides improved sensitivity for identifying infected animals, particularly during the early stages of infection or in cases with low viral load. However, few studies using molecular techniques have been conducted in Guinea, and no published data are currently available regarding the prevalence of canine parvovirus in the urban commune of Labé.

The present study was therefore undertaken to determine the prevalence of canine parvovirus among dogs in the urban commune of Labé using PCR methodology and to identify factors potentially associated with infection. In addition, the study aimed to evaluate dog owners’ knowledge and management practices regarding canine health and vaccination. The findings of this study are expected to contribute to a better understanding of CPV-2 epidemiology in Guinea and to provide baseline data for improving vaccination strategies, owner awareness, and epidemiological surveillance programs.

## Materials and methods

### Study design and study period

A descriptive, analytical, cross-sectional study was conducted between September and November 2024 in the urban commune of Labé, Republic of Guinea, to determine the prevalence of canine parvovirus (CPV-2) and identify factors potentially associated with infection. This study design was selected because it allows the simultaneous assessment of disease occurrence and associated epidemiological factors within a defined population at a specific period.

### Study area and study population

The study was carried out in the urban commune of Labé, located in the Middle Guinea region. This area was selected because of the increasing number of suspected canine parvovirus cases reported by veterinarians and dog owners, as well as the absence of previous epidemiological investigations on CPV-2 in the locality.

The target population consisted of domestic dogs residing in the selected neighborhoods of the urban commune of Labé. Information was also collected from dog owners and breeders through questionnaires addressing dog management practices, vaccination status, and owner knowledge regarding canine parvovirus.

### Inclusion criteria

Dogs were eligible for inclusion in the study if their owners were at least 18 years old and voluntarily agreed to participate in the survey and permit rectal swab sample collection from their animals. Free-roaming, chained, and caged owned dogs were included regardless of sex, age, or breed.

### Clinical evaluation and case definition

Prior to sample collection, all dogs included in the study underwent a clinical examination to identify signs compatible with canine parvovirus infection. Clinical evaluation was performed using standard semiological methods including auscultation, palpation, percussion, and body temperature measurement. Information regarding recent clinical history was also collected from dog owners during the questionnaire survey.

The principal clinical signs investigated included fever, vomiting, lethargy, anorexia, weakness, dehydration, and hemorrhagic diarrhea. Some dogs included in the study were asymptomatic at the time of sampling, whereas others presented clinical manifestations compatible with canine parvovirus infection, particularly bloody diarrhea. Fever was observed in all clinically affected dogs.

Dogs presenting clinical signs suggestive of canine parvovirus infection were considered suspected clinical cases. However, definitive positive cases in this study were established exclusively based on real-time PCR (qPCR) detection of CPV-2 genetic material from rectal swab samples.

### Sampling procedure

A non-probabilistic convenience sampling approach combined with simple random selection of study sites was used. The neighborhoods included in the study were identified using OpenEpi open-source software. Seven neighborhoods were selected within the urban commune of Labé, and approximately fourteen dogs were sampled from each neighborhood.

Within the selected neighborhoods, dogs were recruited according to owner availability and consent. This approach was considered appropriate given the absence of a comprehensive canine population registry and the field conditions encountered during data collection.

### Sample size determination

The sample size was calculated using the formula described by Thrusfield [10] for prevalence studies:

$$\:N=\frac{{\left(1.96\right)}^{2}\times\:P(1-P)}{{d}^{2}}$$

Where:

N = required sample size

P = expected prevalence

d = desired precision

1.96 = standard normal deviate corresponding to a 95% confidence interval

Because no previous prevalence data on canine parvovirus were available in the study area, an expected prevalence of 50% was used to maximize sample size estimation. A precision level of 10% and a confidence interval of 95% were applied.

$$N = \frac{(1.96)^2 \times 0.5(1-0.5)}{(0.1)^2}$$

The minimum estimated sample size obtained was 96 dogs. To improve representativeness and account for potential sampling losses, a total of 102 dogs were ultimately included in the study.

### Data collection procedures

Questionnaire survey.

The questionnaire gathered information regarding:

- Dog age;
- Sex;
- Breed;
- Lifestyle (free-roaming, chained, or caged);
- Vaccination status;
- Owner knowledge of canine parvovirus;
- and animal management practices.

Prior to the main survey, a pre-test of the questionnaire was conducted in selected neighborhoods not included in the final study. This preliminary assessment allowed clarification and adjustment of certain questions to improve comprehension and consistency of responses.

Data collection was performed using KoboCollect software through the KoboToolbox Humanitarian platform.

### Laboratory analysis

#### Sample collection and transport

Rectal swab samples were collected aseptically from all dogs included in the study using sterile swabs. Swabs were immediately placed into sterile transport tubes containing physiological saline solution to preserve viral material during transportation. Samples were labeled individually and transported under cold-chain conditions to the laboratory for molecular analysis.

#### DNA extraction

Viral DNA was extracted from rectal swab samples using the Qiagen DNeasy Blood & Tissue Kit (Qiagen^®^, Germany) according to the manufacturer’s instructions. Extracted DNA was stored at − 20 °C until molecular analysis.

#### Real-time PCR (qPCR) amplification

Detection of canine parvovirus type 2 (CPV-2) DNA was performed using real-time polymerase chain reaction (qPCR) targeting the VP2 gene of canine parvovirus, which is highly conserved and commonly used for molecular diagnosis of CPV-2 infections [4].

PCR methodology was preferred because of its higher sensitivity and specificity compared with rapid fecal antigen detection tests, particularly for detecting low viral loads.

Amplification reactions were performed using CPV-2-specific primers:

Forward primer: 5’-CAGGAAGATATCCAGAAGGA-3’

Reverse primer: 5’-GGTGCTAGTTGATATGTAATAAACA-3’

The expected amplicon size was approximately 583 bp.

Each qPCR reaction mixture contained:

#### PCR master mixo

Specific forward and reverse primers;

Nuclease-free water;

Extracted DNA template (1–5 µL).

The final reaction volume was 25 µL.

Amplification was performed using a real-time thermocycler under the following cycling conditions:

**Table:** 

Step	Temperature	Duration	Number of cycles
Initial denaturation	95 °C	5 min	1
Denaturation	95 °C	30 s	40 cycles
Primer annealing	55 °C	30 s	40 cycles
Extension	72 °C	45 s	40 cycles
Final extension	72 °C	5 min	1

Positive and negative controls were included in each PCR run to ensure the validity and reliability of the amplification process.

Samples presenting cycle threshold (Ct) values ≤ 38 were considered positive according to the laboratory protocol and manufacturer’s recommendations.

The PCR protocol used in this study was designed for the detection of CPV-2 DNA and did not specifically differentiate between CPV-2a and CPV-2b antigenic variants.

The study focused exclusively on molecular detection of CPV-2 DNA and did not include serological analyses for antibody detection or immune status evaluation.

#### Definition of positive cases

Dogs were considered positive when CPV-2 genetic material was detected by PCR amplification. The study focused on molecular detection of viral DNA and did not include serological analysis for the evaluation of antibody levels or previous exposure.

#### Statistical analysis

Data collected through questionnaires and laboratory analyses were entered using KoboCollect software and exported to Microsoft Excel and DATAtab for statistical analysis.

Descriptive statistics were used to summarize prevalence data and epidemiological characteristics of the study population. Associations between CPV-2 infection and potential risk factors, including age, sex, and lifestyle, were evaluated using logistic regression analysis. Odds ratios (OR) and 95% confidence intervals (CI) were calculated, and statistical significance was considered at *p* < 0.05.

## Results

### Overall prevalence results

The study reports an overall prevalence of 10,8%, corresponding to 11 dogs testing positive out of 102. This result indicates active circulation of canine parvovirus in the urban commune of Labé. Although this rate is not extremely high, it reveals a significant level of exposure in the community, particularly in a context where vaccination coverage is low and dog health management is limited. This situation suggests the need to strengthen vaccination and owner awareness.

### Distribution according to the age of dogs

The distribution of positive cases according to dog age is presented in Table 1.

**Table 1:** Distribution according to the age of dogs

Dog age	Number tested	Positive	Prevalence (%)	95% CI (%)
< 1 year	29	2	6.9	0.8–22.8
1–5 years	63	7	11.1	4.6–21.6
6–10 years	10	2	20.0	2.5–55.6
Total	102	11	10.8	5.5–18.5

The prevalence of canine parvovirus varied according to the age of the dogs, ranging from 6.9% in dogs younger than one year to 20.0% in dogs aged 6–10 years. Dogs aged 1–5 years showed an intermediate prevalence of 11.1%.

The highest prevalence observed among older dogs may suggest prolonged cumulative exposure to contaminated environments and infected animals over time. Adult dogs living under free-roaming or poorly managed conditions may remain continuously exposed to CPV-2 circulation within the environment. In contrast, the lower prevalence observed among dogs under one year of age could be related to maternal immunity in some puppies, lower representation of young dogs in the sample, or differences in owner management practices.

However, the wide confidence intervals observed, particularly in the 6–10 years age group (95% CI: 2.5–55.6), indicate limited precision of the estimates, likely due to the relatively small sample size within this category. Overall, these findings suggest possible variation in CPV-2 exposure according to age, although age alone may not be a strong determinant of infection in the study population.

### Distribution by sex

The distribution of positive cases according to sex is presented in Table 2.

**Table 2:** Distribution by sex

Gender of dogs	Number tested	Positive	Prevalence (%)	95% CI (%)
Female	18	1	5.6	0.1–27.3
Male	84	10	11.9	5.9–20.8
Total	102	11	10.8	5.5–18.5

The results presented in Table X show that male dogs had a higher prevalence of canine parvovirus infection (11.9%) compared with female dogs (5.6%). This difference may suggest increased exposure of male dogs to contaminated environments and infected animals. In many African urban settings, male dogs are more frequently allowed to roam freely for guarding, breeding, or territorial behaviors, which may increase their contact with contaminated feces and other infected dogs.

In addition, behavioral and physiological factors may contribute to this difference. Male dogs generally exhibit greater roaming activity and territorial movements, potentially increasing environmental exposure to CPV-2. Some studies have also suggested that hormonal factors, particularly testosterone, may influence immune responses and susceptibility to certain infectious diseases.

However, the wide confidence interval observed among female dogs (95% CI: 0.1–27.3) indicates limited precision of the estimate, mainly due to the small number of females included in the study. Overall, although males appeared more frequently infected than females, the observed difference should be interpreted cautiously because of the relatively small sample size and the overlapping confidence intervals between groups.

### Influence of lifestyle (free-roaming, chained, and caged dogs)

To understand the influence of lifestyle on canine parvovirus, a survey was conducted.

The results are presented in the following table.

**Table 3:** Influence of lifestyle (free-roaming, chained, and caged dogs)

Lifestyle of dogs	Number tested (*n*)	Positive	Prevalence (%)	95% CI (%)
Free-roaming	52	6	11.5	4.3–23.4
Chained	45	5	11.1	3.7–24.1
Caged	4	0	0.0	0.0–60.2
Missing/invalid data	1	—	—	—
Total	102	11	10.8	5.5–18.5

The results presented in Table 3 show that most dogs included in the study were either free-roaming (51.0%) or chained (44.1%), whereas only a small proportion were caged (3.9%). The prevalence of canine parvovirus was slightly higher among free-roaming dogs (11.5%) compared with chained dogs (11.1%), while no positive case was detected among caged dogs.

These findings suggest that free-roaming and chained dogs may be more exposed to contaminated environments, infected feces, and contact with other animals, thereby facilitating CPV-2 transmission. In the urban commune of Labé, dogs frequently move within neighborhoods, markets, and public areas, which may contribute to environmental dissemination of the virus. Chained dogs may also remain exposed through poor hygiene conditions and indirect contact with contaminated materials or animals.

However, the absence of positive cases among caged dogs should be interpreted cautiously because of the very small number of animals included in this category (*n* = 4), as reflected by the wide confidence interval. No statistically significant association was observed between dog lifestyle and CPV-2 infection (*p* > 0.05). These findings suggest that CPV-2 circulation may occur broadly within the environment regardless of dog confinement status, particularly in settings where hygiene measures and preventive veterinary practices remain limited. No statistically significant association was observed between dog lifestyle and CPV-2 infection (*p* = 0.84).

### Geographic distribution of cases

The distribution of positive cases by neighborhood is presented in Table 4.

**Table 4:** Geographic distribution of cases

Neighborhood	Number tested	Positive	Prevalence (%)	95% CI
Labico	18	2	11.1	1.4–34.7
Pounthioun	16	2	12.5	1.6–38.3
Madina	13	1	7.7	0.2–36.0
Safatou 1	13	1	7.7	0.2–36.0
Kouroula	14	2	14.3	1.8–42.8
Tata	14	2	14.3	1.8–42.8
Daka 2	14	1	7.1	0.2–33.9
Total	102	11	10.8	5.5–18.5

The prevalence of canine parvovirus varied across the different neighborhoods of the urban commune of Labé, ranging from 7.1% in Daka 2 to 14.3% in Kouroula and Tata. Intermediate prevalence rates were observed in Labico (11.1%) and Pounthioun (12.5%), while Madina and Safatou 1 recorded lower prevalence values (7.7%).

Although spatial differences were observed between neighborhoods, the wide confidence intervals obtained suggest limited precision of the estimates, likely due to the relatively small sample sizes within each locality. Nevertheless, the higher prevalence observed in Kouroula and Tata may indicate areas with increased viral circulation, potentially associated with higher dog density, uncontrolled roaming, or reduced access to preventive veterinary care.

Overall, these findings suggest heterogeneous spatial distribution of CPV-2 infection within the study area and highlight the importance of strengthening targeted surveillance and prevention measures in high-risk neighborhoods.

### Analysis of risk factors

To determine if age and gender are factors associated with the onset of canine parvovirus, we conducted statistical tests and the results are recorded in the following Table 5.

**Table 5:** Analysis of risk factors

Dog age	Number tested	Positive	Prevalence (%)	95% CI (%)	*p*-value
< 1 year	29	2	6.9	0.8–22.8	—
1–5 years	63	7	11.1	4.6–21.6	—
6–10 years	10	2	20.0	2.5–55.6	—
Total	102	11	10.8	5.5–18.5	0.51

The prevalence of canine parvovirus varied according to the age of the dogs, ranging from 6.9% in dogs less than one year old to 20.0% in dogs aged 6–10 years. Dogs between 1 and 5 years showed an intermediate prevalence of 11.1%.

Although older dogs appeared to present higher prevalence rates, no statistically significant association was observed between age and CPV-2 infection (*p* > 0.05).

The wide confidence intervals obtained, particularly in the 6–10 years age group (95% CI: 2.5–55.6), likely reflect the relatively small sample size within this category and suggest limited precision of the estimates. Overall, these findings suggest possible variation in CPV-2 exposure according to age; however, age was not identified as a significant risk factor in the present study.

**Fig. 1:**
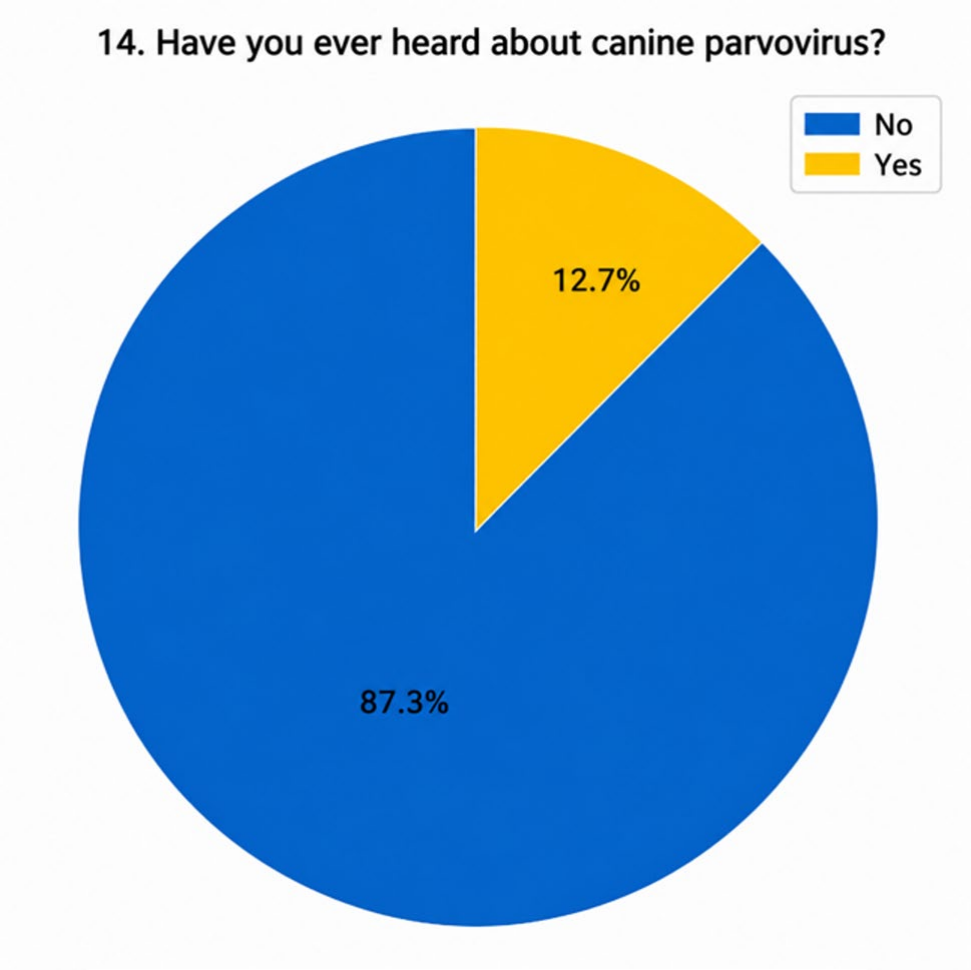
Awareness of canine parvovirus among respondents

Figure 1 illustrates the level of awareness of canine parvovirus among respondents in the urban commune of Labé. The distribution of participants according to whether they had previously heard about canine parvovirus is presented below.

Figure 1 shows that most respondents had little or no knowledge of canine parvovirus in the urban commune of Labé. This low level of awareness may be linked to limited veterinary public education, poor access to veterinary services, absence of routine vaccination programs, and the low prioritization of companion animal health in Guinea. In addition, dogs are often kept mainly for security or hunting purposes, with limited preventive healthcare practices. These findings highlight the need to strengthen awareness campaigns and improve veterinary preventive services in the study area.

## Discussion

### Overall prevalence of canine parvovirus

The present study revealed an overall prevalence of 10.8% for canine parvovirus (CPV-2) in the urban commune of Labé, confirming active viral circulation within the study area. This prevalence indicates that CPV-2 remains an important canine health concern in Guinea, particularly in a context characterized by limited preventive veterinary measures and low public awareness regarding canine infectious diseases. Although the prevalence observed in the present study was lower than the 46% reported by Castillo et al., [1] in Chile, differences between studies may be related to variations in diagnostic methods, sample types, epidemiological conditions, and characteristics of the dog populations investigated.

The persistence of CPV-2 circulation in Labé may be associated with several contextual factors commonly encountered in low-resource African settings, including insufficient vaccination practices, limited access to veterinary healthcare services, uncontrolled dog roaming, and poor owner awareness regarding canine diseases. In Guinea, canine parvovirus is not considered a major priority disease in veterinary public health programs, and vaccination against CPV-2 is not routinely practiced. Consequently, many dog owners remain unfamiliar with the disease, its modes of transmission, and preventive measures.

The predominance of free-roaming and chained dogs observed in the present study may also contribute to environmental contamination and viral persistence. Because CPV-2 is highly resistant in the environment and can survive for prolonged periods in contaminated feces, poor hygiene conditions and unrestricted dog movement may facilitate viral transmission between animals.

In addition, the use of real-time PCR (qPCR) allowed sensitive molecular detection of CPV-2 genetic material and confirmed active viral circulation among dogs in the study area. The present study therefore provides valuable baseline epidemiological data on canine parvovirus in Guinea, where molecular information regarding CPV-2 remains scarce. These findings highlight the importance of strengthening vaccination programs, improving owner awareness, promoting responsible dog management practices, and reinforcing epidemiological surveillance to reduce CPV-2 transmission and associated canine health losses.

### Distribution according to age of dogs

The prevalence of canine parvovirus varied according to age group, with the highest prevalence observed among dogs aged 6–10 years (20.0%), followed by dogs aged 1–5 years (11.1%), whereas dogs younger than one year showed the lowest prevalence (6.9%). Although canine parvovirus is generally considered more common in puppies because of the immaturity of their immune system, infections in adult dogs may also occur in settings characterized by poor vaccination practices and persistent environmental contamination.

The higher prevalence observed among older dogs in the present study may reflect prolonged cumulative exposure to contaminated environments and infected animals over time. In the urban commune of Labé, many dogs live under free-roaming or chained conditions, which may increase repeated exposure to contaminated feces and environmental sources of CPV-2. In addition, chronic parasitic infections, malnutrition, or other immunosuppressive conditions commonly encountered in low-resource environments may contribute to increased susceptibility among adult dogs.

The lower prevalence observed among dogs younger than one year could be associated with maternal antibody protection in some puppies, differences in owner management practices, or the relatively small number of young animals included in the study. Similar findings were reported by Chabchoub et al., [2], who also found no significant association between age and CPV-2 infection. In contrast, Oliveira et al., [8] and Khaled and Souleyman [6] reported higher infection rates among puppies.

However, no statistically significant association was identified between age and CPV-2 infection (*p* = 0.51). The wide confidence intervals observed, particularly among dogs aged 6–10 years, likely reflect the relatively small sample size within this category and indicate limited precision of the estimates. Therefore, although variations in prevalence were observed between age groups, age alone could not be identified as a significant determinant of CPV-2 infection in the present study.

### Distribution according to sex

Male dogs presented a higher prevalence of CPV-2 infection (11.9%) compared with females (5.6%). Similar observations were reported by Rousselle [9], who described a predominance of infection among male dogs. This difference may be explained by behavioral and management factors rather than biological sex alone.

In many African urban settings, male dogs are more frequently allowed to roam freely for guarding, breeding, or hunting purposes, increasing their exposure to contaminated environments and contact with infected animals. Increased mobility and social interactions among male dogs may therefore contribute to greater viral exposure.

Despite the higher prevalence observed among males, the association between sex and infection was not statistically significant (*p* = 0.43). This finding is consistent with Chabchoub et al., [2], who also reported no significant association between sex and canine parvovirus infection. The wide confidence intervals observed in the present study suggest limited precision of the estimates, likely related to the relatively small sample size.

### Influence of lifestyle

The analysis of dog lifestyle showed that most dogs included in the study were either free-roaming (51.0%) or chained (44.1%), whereas only a small proportion were permanently caged (3.9%). The prevalence of canine parvovirus was slightly higher among free-roaming dogs (11.5%) compared with chained dogs (11.1%), while no positive case was detected among caged dogs. However, no statistically significant association was observed between dog lifestyle and CPV-2 infection (*p* > 0.05).

These findings suggest that environmental exposure may contribute to CPV-2 circulation in the urban commune of Labé. Free-roaming dogs are generally more exposed to contaminated environments, infected feces, markets, streets, and contact with other animals, thereby increasing the risk of viral transmission. Similarly, chained dogs may remain exposed through contaminated surroundings, poor hygiene conditions, indirect contact with infected animals, or contaminated materials transported between households.

The absence of positive cases among caged dogs could indicate reduced environmental exposure due to stricter confinement practices. However, this result should be interpreted cautiously because only four dogs belonged to this category, resulting in a wide confidence interval and limited statistical representativeness. In addition, the relatively small number of positive cases detected in the study may have reduced the statistical power required to demonstrate a significant association between lifestyle and infection.

Overall, these findings indicate that dog management practices and environmental contamination may contribute to CPV-2 circulation in the study area. Similar observations were reported by Khaled and Souleyman [6], who noted increased exposure to canine infectious diseases among dogs living under free or semi-confined conditions. The present study therefore provides valuable baseline epidemiological information on the relationship between dog lifestyle and CPV-2 circulation in Guinea, where data on canine infectious diseases remain limited. These results may contribute to the development of preventive strategies aimed at improving dog confinement practices, environmental hygiene, and owner awareness in order to reduce viral transmission.

### Geographic distribution of cases

The prevalence of canine parvovirus varied between neighborhoods, ranging from 7.1% in Daka 2 to 14.3% in Kouroula and Tata. This heterogeneous spatial distribution suggests the existence of localized transmission areas within the urban commune of Labé.

Higher prevalence rates observed in Kouroula, Tata, and Pounthioun may be associated with increased dog density, uncontrolled roaming, poor sanitation, and reduced access to veterinary care. Similar spatial variability has been reported in Brazil by Oliveira et al., [8] and in Tunisia by Chabchoub et al., [2], where local environmental and management conditions influenced viral circulation.

These findings underline the importance of targeted prevention strategies in high-risk neighborhoods, including vaccination campaigns, owner education, and improved management of free-roaming dogs.

### Analysis of risk factors

Logistic regression analysis did not identify statistically significant associations between CPV-2 infection and the investigated demographic factors, including age and sex (*p* > 0.05). Although male dogs and older animals showed higher odds ratios, the confidence intervals remained wide, suggesting limited precision of the estimates.

The lack of significant associations may be explained by the relatively small sample size and the limited number of positive cases identified during the study. Similar observations were reported by Chabchoub et al., [2]. In contrast, Oliveira et al., [8] and other authors identified younger age as a significant risk factor due to immune immaturity and incomplete vaccination.

Overall, the findings suggest that CPV-2 exposure in the study area is likely influenced by multiple interacting environmental and management factors rather than by a single demographic characteristic.

### Owners’ knowledge and practices

Most respondents demonstrated limited awareness of canine parvovirus. This finding reflects the low level of veterinary public education and the limited dissemination of information regarding companion animal diseases in Guinea. Unlike rabies, canine parvovirus receives little attention in veterinary extension programs and public awareness campaigns. In Guinea, canine parvovirus is not considered a major priority disease in veterinary public health programs, which may further explain the low level of owner awareness observed in this study.

In many households, dogs are primarily kept for security, hunting, or traditional purposes rather than as companion animals receiving preventive healthcare. In addition, financial limitations, restricted access to veterinary clinics, and low educational levels among some dog owners may contribute to poor knowledge regarding canine infectious diseases and vaccination practices.

These findings are consistent with those reported by Zaoro [11], who also observed limited owner awareness regarding canine parvovirus. Improving public awareness and strengthening preventive veterinary education could contribute significantly to reducing viral circulation and improving canine health management in the study area.

## Conclusion

This study confirmed the circulation of canine parvovirus (CPV-2) in the urban commune of Labé, Guinea, with an overall molecular prevalence of 10.8% detected by PCR analysis. The findings demonstrate that CPV-2 remains an important canine health concern in the study area, particularly in a context characterized by low awareness of the disease, absence of routine vaccination practices, limited veterinary preventive services, and frequent free-roaming dog management.

Higher prevalence rates were observed among male dogs, adult dogs, and free-roaming animals; however, no statistically significant association was identified between CPV-2 infection and the investigated demographic factors. These findings suggest that viral transmission may be influenced by multiple environmental and management-related factors rather than by age or sex alone.

The study also revealed limited owner knowledge regarding canine parvovirus and inadequate preventive practices. In Guinea, canine parvovirus remains poorly known among dog owners, and vaccination against the disease is rarely practiced. This situation may contribute to the persistence and spread of the virus within the canine population.

Despite certain limitations, including the relatively small sample size and the absence of serological analyses, this study provides important baseline epidemiological data on canine parvovirus in Guinea. The results highlight the need to strengthen epidemiological surveillance, improve owner awareness, promote responsible dog management practices, and increase access to preventive veterinary healthcare and vaccination programs.

Further large-scale molecular and serological investigations are recommended to better understand CPV-2 circulation dynamics, characterize circulating viral variants, and support the development of effective prevention and control strategies in Guinea.

### Strengths and Limitations of the Study

This study represents one of the first molecular epidemiological investigations of canine parvovirus (CPV-2) in the urban commune of Labé, Guinea. The use of PCR improved the accuracy of viral detection and confirmed active CPV-2 circulation in the study area. In addition, the combination of laboratory analyses and epidemiological surveys provided useful information on owner awareness and dog management practices.

However, some limitations should be considered, including the relatively small sample size, reliance on owner-reported vaccination status, absence of serological analyses, and the inability of the PCR protocol to differentiate CPV-2 variants. Moreover, the possibility of false-positive PCR results related to contamination cannot be entirely excluded.

Despite these limitations, the study provides important baseline data for future molecular surveillance and control strategies against canine parvovirus in Guinea.

## Data Availability

The datasets utilized and/or examined in the present study are not publicly accessible but can be obtained from the corresponding author upon reasonable request.

## Abbreviations

CPV-2: Canine Parvovirus type 2
CPV-2a / CPV-2b: Antigenic variants of Canine Parvovirus type 2
CPV: Canine Parvovirus
PCR: Polymerase Chain Reaction
DNA: Deoxyribonucleic acid
OR: Odds Ratio
CI: Confidence Interval
ISSMV: Higher Institute of Veterinary Science and Medicine
